# Outcomes of Universal Access to Antiretroviral Therapy (ART) in Georgia

**DOI:** 10.1155/2011/621078

**Published:** 2011-02-21

**Authors:** Tengiz Tsertsvadze, Nikoloz Chkhartishvili, Lali Sharvadze, Natia Dvali, Otar Chokoshvili, Pati Gabunia, Akaki Abutidze, Kenrad Nelson, Jack DeHovitz, Carlos del Rio

**Affiliations:** ^1^Infectious Diseases, AIDS and Clinical Immunology Research Center (IDACIRC), 16 Al. Kazbegi Avenue, Tbilisi 0160, Georgia; ^2^Faculty of Medicine, Tbilisi State University, Tbilisi, Georgia; ^3^Department of Epidemiology, Johns Hopkins Bloomberg School of Public Health, 615 North Wolfe Street, W6508, Baltimore, MD 21205, USA; ^4^Department of Medicine, SUNY Downstate Medical Center, 450 Clarkson Avenue, Box 1240 Brooklyn, NY 11203, USA; ^5^Hubert Department of Global Health, Rollins School of Public Health of Emory University, 1518 Clifton Road, NE Room 754, Atlanta, GA 30322, USA

## Abstract

Since 2004, Georgia achieved universal access to free antiretroviral therapy (ART). A retrospective cohort study was conducted to evaluate the outcomes of Georgia's ART program. The study included adult patients enrolled in the ART program from 2004 through 2009. Of 752 patients, 76% were men, 60% were injection drug users (IDU), 59% had a history of an AIDS-defining illness, and 53% were coinfected with hepatitis C. The median baseline CD4 cell count was 141 cells/mm^3^. During followup, 152 (20%) patients died, with the majority of deaths occurring within 12 months of ART initiation. Mortality was associated with advanced immunodeficiency or the presence of incurable disease at baseline. Among patients remaining on treatment, the median CD4 gain was 216 cell/mm^3^ and 86% of patients had viral load <400 copies/ml at the last clinical visit. The Georgia ART program has been successful in treating injection drug users infected with HIV.

## 1. Introduction

The advent of highly active antiretroviral therapy in the mid 1990s fundamentally altered the natural history of HIV infection in industrialized countries, resulting in dramatic reduction in AIDS-related morbidity and mortality [1–3]. However, for many years access to HAART in low- and middle-income countries was limited, primarily due to the price of antiretroviral drugs that made them beyond the reach of most patients. The momentum generated by the World Health Organization's “3 by 5” strategy resulted in considerable progress in expanding access to lifesaving treatment in resource-limited countries [4]. Analyses of HIV treatment programs in low- and middle-income countries have already shown positive outcomes in terms of response to therapy and declining mortality [5–11].

Georgia, Armenia, and Azerbaijan make up a group of former Soviet republics known as the South Caucasus Republics. With the fall of the Soviet Union and the independence of all these republics in 1991, much of the social structure supporting health care became increasingly dysfunctional and the system of national healthcare that held a high standard for all Soviet citizens fell into disarray as economies crashed and conflicts within and between the countries disrupted services and infrastructure. Each one of these countries has its unique set of problems and issues, but, in general, their declining economic situations, coupled with rising drug use and commercial sex work, and their geographic proximity to Russia and Ukraine, countries with emerging epidemics, make the South Caucasus a region ripe for the spread of HIV. 

Georgia is located at the juncture of Eastern Europe and Western Asia and is bordered by Russia, Azerbaijan, Armenia, and Turkey. In 2009, the population of Georgia was estimated to be 4,615,807 in a territory of 26,900 sq miles. The GDP per capita in 2009 was approximately $4,300 US ranking 103 in the world, and the GINI coefficient was 40.8 ranking 59th in the world. Georgia regained independence in August of 1991, followed by a two-year civil war that led to large population migration with internal displacement, severe poverty, a dysfunctional economy, and disruption of many national services including healthcare. The country was stabilized in 1995, but the dire economic position of the Georgian government necessitated conversion of the state-funded health care system to a market-driven system. Nearly half of all Georgians are now forced to forego professional medical care when sick, opting instead for advice from friends and relatives, traditional medicine, or no care [12].

The first case of HIV in Georgia was reported in 1989. As of December 31, 2009 a cumulative 2,236 HIV cases had been reported. Among them 1,151 persons developed AIDS and 479 died. Males constitute the majority (74%), of the reported HIV/AIDS cases with injection drug use (IDU) responsible for HIV acquisition among nearly 60% of all reported cases. The estimated HIV prevalence in Georgia is less than 0.1%. Antiretroviral drugs have been available in the country since 1990s, but the access was limited only to those able to afford them. Since 2004, through support from the Global Fund, Georgia became the first country among the newly independent states (NIS) of Eastern Europe to achieve universal access to antiretroviral therapy (ART). This major achievement has been acknowledged in the joint WHI/UNAIDS/UNICEF report on universal access [4]. ART coverage estimation is based on standard WHO methodology using SPECTRUM projection software. According to this report, Georgia is among few low- and middle-income countries with highest attainable coverage. The objective of this study is to evaluate outcomes of Georgia's free ART program.

## 2. Methods

### 2.1. Study Design

A retrospective cohort study was conducted at the Infectious Diseases, AIDS and Clinical Immunology Research Center (IDACIRC), in Tbilisi, Georgia, which is the country's referral institution for HIV/AIDS diagnosis, treatment, and care. The study population included all adult (age ≥18 year) patients enrolled in the ART program from 2004 through September 30 2009, who started therapy for at least six month prior to April 1, 2010.

### 2.2. Description of the ART Program in Georgia

The National ART Program is coordinated by the IDACIRC, and antiretroviral drugs are dispensed in the IDACIRC clinic in the capital city of Tbilisi as well as at three affiliated regional centers in the cities of Kutaisi, Batumi, and Zugdidi. HIV infected persons are identified through state and donor funded HIV testing and counseling (HTC) services and screening programs. All persons with positive screening test results are referred to IDACIRC for confirmatory testing and if confirmed are initially assessed at IDACIRC. Patients have the option to continue clinical care either at central or regional levels.

Provision of therapy is governed by the National HIV/AIDS Treatment and Care guidelines developed based on the protocols of WHO as well as the guidelines of major Western countries [13–16]. The first guidelines were developed in 2004 and have been regularly updated thereafter. At the time that the patients included in this analysis were started on ART, treatment was recommended when the CD4 cell count was ≤200/mm^3^ or if the patient had an AIDS defining illness. ART was also recommended at CD4 cell count of ≤350/mm^3^, based on the CD4 cell decline rate, a high HIV-1 viral load, and coinfection with viral hepatitis. Currently, steps are being taken towards implementation of the recommendation to initiate treatment in all patients with CD4 cell count of ≤350/mm^3^. 

Identification of patients in need of treatment is based on following those who don't qualify for therapy every 3-4 months and monitoring the CD4 cell count and HIV viral load. This allows for the timely identification of those in need of treatment.

The recommended initial regimen consists of two nucleoside reverse transcriptase inhibitors (NRTI) and one nonnucleoside reverse transcriptase inhibitor (NNRTI). A ritonavir (r) boosted protease inhibitor (PI) is recommended in cases when an NNRTI cannot be prescribed. Currently tenofovir (TDF) + emtricitabine (FTC), zidvudine (AZT), or abacavir (ABC) + lamivudine (3TC) are used for the NRTI component of initial regimen. Stavidine (d4T) is no longer recommended after the 2007 revision of National Guidelines; however the drug is reserved for short-term use for situations when AZT, ABC, or TDF cannot be used because of severe toxicity. Since 2008, patients are tested for HLA B∗5701 before starting on an ABC-containing regimen. Efavirenz (EFV) is the preferred NNRTI with nevirapine (NVP) being recommended as an alternative to EFV. 

Selection of subsequent regimens in treatment-experienced patients is based on the drug resistance profile with the goal of providing patients with at least two, and preferably three, fully active drugs. In addition to boosted PIs (ATV/r, DRV/r, FPV/r, LPV/r), new classes of drugs are also now available for highly treatment-experienced patients, such as the integrase strand transfer inhibitor raltegravir (RAL), the fusion inhibitor enfuvirtide (ENF), and the CCR5 antagonist maraviroc (MVC). Recently, the new NNRTI etravirine (ETV) has also become available.

As per the Georgian National guidelines, the standard of ART monitoring relies upon laboratory monitoring of CD4 count, HIV-1 viral load, and development of resistance based on a resistance-genotype detection when indicated. Virological failure is defined as confirmed plasma HIV-1RNA >400 copies/ml 6 months after starting therapy or plasma HIV-1 RNA >50 copies/ml 12 months after starting therapy in a patient who is on potent ART. 

Georgia was the first NIS country to introduce genotypic resistance testing in 2005 into routine clinical practice. HIV drug resistance testing is used to guide treatment decisions during virologic failure and to make decisions as to the most effective subsequent regimen.

Special attention is paid to adherence to therapy as an important determinant of treatment success. A program to promote and maintain antiretroviral adherence has been developed that includes maintenance of an adherence diary, pill identification by shape and color, patient self-report about the medication intake in the preceding 3- to 7-day period and medication refill using pharmacy records. In addition, to improve adherence, mobile units to deliver home-based adherence support operate countrywide.

### 2.3. Laboratory Assays

Plasma HIV-1 RNA levels were initially measured using Amplicor HIV-1 Monitor test, version 1.5 (Roche Molecular Diagnostics, Germany), with lower limit of detection of 400 copies/ml. Since 2006, the real-time PCR assay COBAS TaqMan HIV-1 test (Roche Molecular Diagnostics, Germany) has been in use with a lower limit of detection of 40 copies/ml.

Determination of CD4+ cell count is based on the single-platform immunophenotyping technique using the FACSCalibur flow cytometer (Becton-Dickinson, USA) with four-color direct immunofluorescence reagent MultiTEST CD3/CD8/CD45/CD4.

For genotypic resistance testing, the TruGene HIV-1 Genotyping Kit was employed according to the manufacturer's instructions using OpenGene DNA Sequencing System (Siemens Medical Solutions Diagnostics, Germany). The Guidelines Rules version 14.0 and Stanford University algorithm (http://hivdb.stanford.edu/) were used for resistance interpretation. Mutations listed by the International AIDS Society-USA Panel were considered [17].

### 2.4. Statistical Analysis

Data were obtained from the National HIV/AIDS electronic database, operated by IDACIRC. The database contains information on all reported HIV cases, including demographic, epidemiological, clinical, and laboratory data. Information on all patients initiating HAART from 2004 through 2009 was extracted. Observations were censored as of April 1, 2010. Descriptive statistics were performed to assess distribution of covariates. Normality of continuous variables was evaluated using Q-Q plots. Kaplan-Meier product-limit estimator method was used to assess probability of survival and probability of virological failure. Predictors of mortality were evaluated in multivariate Cox proportional hazards model. The proportional hazards assumption was tested and was met for the final model. All tests were two-sided at a significance level of 0.05. Statistical analyses were performed using SAS v 9.2 (SAS Institute, Cary, NC, USA).

### 2.5. Ethical Approval

Study was approved by Institutional Review Board (IRB) of the Infectious Diseases, AIDS and Clinical Immunology Research Center. The study was based on information routinely collected as part of the standard of clinical care of HIV infected individuals.

## 3. Results

**Figure 1:**
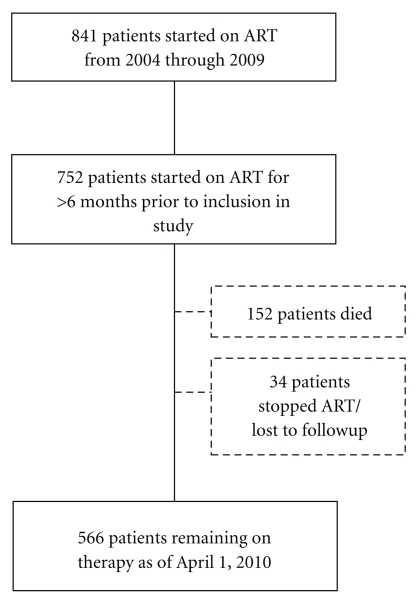
Cohort profile.

Since 2004, over 1,800 patients have been seen for HIV clinical care at the IDACIRC and of these, 841 adults met the treatment initiation criteria and were enrolled in the HAART program (through December 2009). This analysis includes 752 adult patients who had started therapy at least six months prior to inclusion in the study (Figure 1).

Table 1 summarizes the baseline characteristics of these 752 patients. Their median age was 37 years (Interquartile range [IQR] 33–43), and 75% were men. The most common mode of HIV transmission was injection drug use (IDU)- 60%, followed by heterosexual contact (35%). The median CD4 cell count at treatment initiation was 141 cells/mm^3^ (IQR 76–208), with approximately one third of patients having a CD4 cell count less than 100 cells/mm^3^. Median viral load was 5.4 log_10_ copies per ml (IQR 4.8–5.8). Fifty-nine percent of patients had a history of an AIDS defining illness (ADI), with 32.4% having a history of active tuberculosis. More than half of the patients had antibodies against Hepatitis C virus (HCV), 8% of patients had evidence of chronic Hepatitis B infection, and 6% had dual infection with HCV and HBV. Fourteen percent of patients had cirrhosis. Almost 3% of patients had a malignancy.

All but 5 patients were started on an NNRTI-based regimen, most frequently with EFV (82.9%). AZT + 3TC was the most common NRTI component of the first ART regimen (53.0%), followed by ABC + 3TC (27.2%) and d4T + 3TC (18.3%). After the 2007 revision of the national HIV/AIDS Treatment and Care guidelines, all patients on d4T were switched to AZT, ABC, or TDF.

The median duration of followup was 24 months (IQR 10–45 months). During followup, 152 (20.2%) patients died and 34 (4.5%) patients self-discontinued ART/were lost to follow-up, with overall retention rate of 84%. 

**Figure 2:**
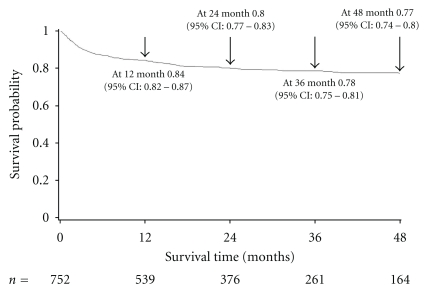
Kaplan-Meier curve of survival probability.

Kaplan-Meier estimates of survival probability were 0.84 (95% CI: 0.82–0.87), 0.80 (95% CI: 0.77–0.83), 0.78 (95% CI: 0.75–0.81), and 0.77 (95% CI: 0.74–0.80) at 12, 24, 36, and 48 months, respectively (Figure 2). Of 152 patients, 115 (75%) died within 12 months of HAART initiation. Median time to death was 3 months (IQR 1–10). Most common causes of death were tuberculosis (34 cases, 22%) and end stage liver disease (29 cases, 19%). Eleven percent of patients died due to incurable malignancies at baseline, including Kaposi's sarcoma, HIV-related lymphomas, invasive cervical cancer, lung cancer, and breast cancer. Other causes of death included cryptococcal meningitis, cardiovascular diseases, wasting syndrome, and infectious diseases of unknown origin.

Factors associated with mortality were assessed in a multivariate Cox proportional hazards model. The following baseline factors were associated with death: male gender (Hazard ratio [HR] 1.96, 95% CI 1.19–3.24), CD4 cell count <100 cells/mm^3^ (HR 2.06, 95% CI 1.48–2.87), history of an AIDS-defining illness (HR 2.01, 95% CI 1.36–2.96), cirrhosis (HR 1.95, 95% CI 1.36–2.81). Other covariates did not show statistical significance in multivariate analysis (Table 2). Presence of active TB was fitted into the multivariate model independently from other ADIs; it showed only active TB to be marginally significant with an HR of 1.40 (95% CI 1.01–1.97).

Among 566 patients still on ART, the median gain of CD4 cells was 216 cells/mm^3^ (IQR 112–348) and 487 (86%) patients had an HIV-1 viral load measurement of <400 copies/ml at their last clinical visit. Data on medication refill adherence were available starting from 2007; the refill adherence rates were 85% in 2007, 83% in 2008, and 92% in 2009.

**Figure 3:**
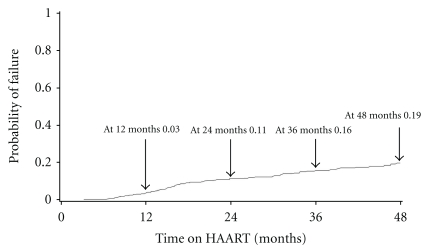
Kaplan-Meier curve of virological failure probability.

Eighty-two patients (10.9%) experienced virological failure. All of them have been tested for the presence of drug resistance virus while on a failing regimen. Median time to virologic failure was 16 months (IQR: 10–28 months). Kaplan-Meier probability of failure at 12, 24, 36, and 48 months were 0.04, 0.11, 0.16, and 0.19, respectively (Figure 3). Of 82 patients, 65 (79.3%) had mutations consistent with antiretroviral resistance. Resistance to a single drug class (NRTI or NNRTI) was found in 4 (4.9%) patients; dual-class drug resistance to NRTI and NNRTI occurred in 61 (74.4%) patients. 

**Figure 4:**
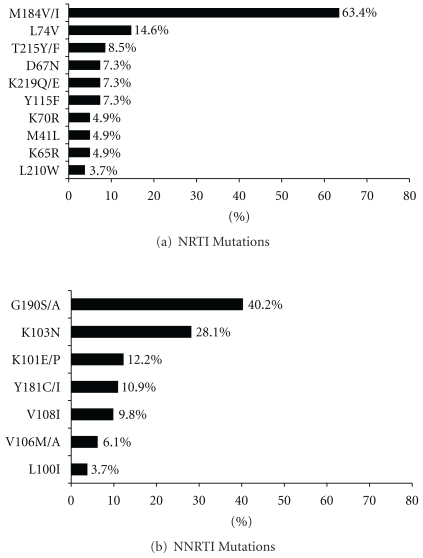
Frequency of resistant mutations in reverse transcriptase gene.

The frequency of drug resistance mutations in the reverse-transcriptase (RT) gene is shown in Figure 4. The most commonly detected NRTI mutation was M184V/I (63.4%). The frequency of thymidine analogue mutation (TAM) (M41L, D67N, K70R, L210W, T215Y/F, and K219Q/E) was relatively low, with only 14 (17.1%) patients having virus with any TAM. Only five (6.1%) patients had viruses with ≥3 TAMs. G190S/A was the most frequent NNRTI mutation (40.2%), followed by K103N (28.1%). No major PI mutations were detected.

All patients with drug resistant viruses were switched to second line regimens with boosted PIs. At present, of 56 patients remaining on second-line therapy, 61%, 21%, 11%, and 7% receive LPV/r, ATV/r, DRV/r, and FPV/r based regimens respectively. Three patients are on salvage regimens, consisting of ENF plus optimized background therapy.

## 4. Discussion

We report the outcomes of the universal ART access program in Georgia. The program builds upon the successful training of young clinicians and scientists who started in their training in the care of HIV/AIDS patients in the 1990s with the goal of providing high quality clinical care in an environment that promotes research. The capacity to provide universal access to ART has been further strengthened through the support of the Global Fund. Under this framework, the approach to ART provision taken by Georgia has been designed to maximize effectiveness of the intervention.

The IDACIRC mandate—as the lead agency for the provision of all HIV care activities in the country—ensures universal access and high retention on therapy, with less than 5% drop-out rate. In addition, a coordinated approach to ART monitoring, including availability of laboratory monitoring (CD4 cell counts, HIV-1 viral load and HIV genotypic resistance testing), and adherence counseling contributes to the achievement of optimal outcomes. Establishment of mobile units for providing home-based adherence support and monitoring resulted in improvement of medication refill adherence from 83% in 2008 to 92% in 2009. Good immunological and virologic responses seen in our cohort also serve as an evidence of good overall adherence. 

The probability of virological failure in our study was similar to that reported from developed countries [18, 19]. In contrast to most other resource-limited countries, where treatment monitoring is limited to clinical findings and changes in CD4 cell counts [20], viral load and drug resistance testing are performed in Georgia as part of the standard of care. Earlier, we have shown feasibility and effectiveness of routine use of these laboratory tools in early identification of patient failing on ART and in improving clinical outcomes in patients with drug resistant viruses [21]. Our current analysis corroborates previous findings: overall the small number of mutations and low frequency of TAMs is suggestive of shorter exposures to failing regimens among our patients, thus preventing the opportunity for mutations to accumulate. 

Interestingly, the most common NNRTI mutation detected in our study was G190S/A but not K103N. It is possible that G190S/A is the favoured NNRTI mutation in HIV subtype A1—which is the most common circulating strain in Georgia [22]. Further investigation of clade-specific resistance pathways is needed to provide more detailed picture, which may have important implications for the clinical management of infection with HIV subtype A1.

An important challenge that remains to be addressed and that has been identified through this study is the early mortality of patients who start ART in Georgia. As shown in our analysis, the highest mortality rate was observed within the first year of ART initiation, with 75% of all deaths occurring in the first 12 months. Our results are consistent with findings from other resource-constrained countries reporting increased risk of mortality early after starting ART [23–26]. In our study, the majority of deaths were due to either advanced HIV disease, as evidenced by severe immunodeficiency due to the history of an ADI or the presence of incurable non-AIDS defining conditions. At present a large proportion of persons in Georgia continue to enter health care very late in the course of their chronic HIV infection, often resulting from missed opportunities to test for HIV in healthcare settings. This is particularly evident among male IDUs, whose HIV disease is further complicated by other comorbidities, such as hepatitis C. This also explains the twofold increased risk of dying in men compared to women. Expanding HTC services, especially among most-at-risk populations, is the key for ensuring earlier HIV diagnosis and treatment initiation.

The most common causes of death in our cohort were TB and end stage liver disease, together accounting for more than 40% of all deaths. Multivariate analysis showed a strong association between cirrhosis and death, emphasizing the problem of coinfection with viral hepatitis, especially with hepatitis C. Dually infected patients virtually have no access to anti-HCV therapy because of prohibitively high costs. Due to this fact, HIV/HCV coinfected patients die from hepatic disease despite the successful antiretroviral therapy. The high prevalence of coinfection with HCV among patients with HIV in Georgia may prevent the full realization of the benefits of ART, and may compromise the cost-effectiveness of ART. Recent studies have shown that the availability of ART does not decrease the risk of mortality among patients with a dual infection with HIV and HCV [27]. Moreover, HIV/HCV coinfected IDUs have been shown to be at 7-fold increased risk of dying from end stage liver disease compared to HCV monoinfected IDUs [28].

Another important challenge is HIV/TB coinfection. The high prevalence of this coinfection in our cohort was not a surprise given the overall high burden of TB in the country [29]. However, the high mortality from TB is of particular concern. Collaboration between the HIV/AIDS and TB services in Georgia is excellent, and all patients have free access to both TB treatment and ART. However, it is clear that the outcomes are not as good as we would have predicted. One possible challenge is limited information on TB drug susceptibility as these data were only available for very few patients. Taking into account the high prevalence of multidrug resistant TB in Georgia (7% in newly diagnosed and 27% in previously treated patients [30]), TB drug resistance may have contributed to excess death from TB similar to that experienced in other countries [31].

As with all studies, limitations should be mentioned. First, the study was based on data available in national HIV/AIDS database. Adverse events of ART are carefully monitored, the data are not entered into electronic database, and therefore we were not able to determine contribution of adverse events to outcomes of interest. A further limitation is that the only baseline information on drug abuse was available and this precluded us from assessing the impact of current drug use on outcomes.

In summary, the HIV epidemic in Georgia has entered a new phase. ART has been successfully introduced in Georgia, including in IDU population. The next stage is to maximize and sustain the benefits of ART. Mortality can be substantially reduced by improving earlier HIV diagnosis and initiation of ART. The comprehensive program for the identification and treatment of patients with HIV/AIDS in Georgia, a relatively low income country, has been quite successful in limiting premature mortality and morbidity from this epidemic disease. Increased efforts and new strategies are needed to effectively address the intersecting HIV/TB and HIV/HCV epidemics and their resulting mortality.

## Figures and Tables

**Table 1:** Baseline characteristics.

Characteristic	*n* = 752
Age, median years (IQR)	37 (33–43)
Gender, *n* (%)	
Male	570 (75.8)
Female	182 (24.2)
Mode of transmission, *n* (%)	
Injection drug use	454 (60.4)
Heterosexual contact	265 (35.2)
Male-to-male sex	17 (2.3)
Blood recipient	6 (0.8)
Other/not identified	10 (1.3)
CD4 cell count, median cells/mm^3^ (IQR)	141 (76–208)
HIV RNA load, median log_10_ copies/ml (IQR)	5.4 (4.8–5.8)
AIDS defining illness, *n* (%)	446 (59.3)
Malignancy, *n* (%)	22 (2.9)
Liver related diseases, *n* (%)	
Anti-HCV+	397 (52.8)
HbsAg+	63 (8.4)
Anti-HCV+/HbsAg+	47 (6.3)
Cirrhosis	104 (13.8)
Initial HAART regimen, *n* (%)	
AZT + 3TC + EFV	310 (41.2)
AZT + 3TC + NVP	86 (11.4)
AZT + 3TC + LPV/r	3 (0.4)
ABC + 3TC + EFV	187 (24.9)
ABC + 3TC + NVP	15 (2.0)
ABC + 3TC + LPV/r	2 (0.3)
TDF + FTC + EFV	9 (1.2)
TDF + FTC + NVP	2 (0.3)
d4T + 3TC + EFV	126 (16.7)
d4T + 3TC + NVP	12 (1.6)

**Table 2:** Cox proportional hazards model analysis of factors associated with death.

	Univariate analysis, HR (95% CI)	Multivariate analysis, HR (95% CI)
Age		
<35 years	1	
>35 years	1.52 (1.07–2.16)	NS
Gender		
Female	1	1
Male	2.14 (1.38–2.32)	1.96 (1.19–3.24)
Mode		
Non-IDU	1	
IDU	1.99 (1.39–2.86)	NS
CD4		
>100 cells/mm^3^	1	1
<100 cells/mm^3^	2.50 (1.82–3.45)	2.06 (1.48–2.87)
Viral load		
<100,000 copies/ml	1	
>100,000 copies/ml	1.67 (1.15–2.45)	NS
AIDS defining illness		
No	1	1
Yes	2.69 (1.90–3.81)	2.01 (1.36–2.96)
Anti-HCV		
Negative	1	
Positive	1.40 (1.01–1.93)	NS
HbsAg		
Negative	1	
Positive	1.77 (1.10–2.83)	NS
Cirrhosis		
No	1	1
Yes	2.05 (1.24–3.40)	1.95 (1.36–2.81)

HR = Hazard ratio, CI = Confidence interval, NS = Not significant.
